# Quality Improvement: Increasing Conversations About Nicotine Replacement Therapy on a General Adult Inpatient Ward

**DOI:** 10.1192/bjo.2026.11434

**Published:** 2026-06-30

**Authors:** Holly Melvin, Georgia Penny

**Affiliations:** Merseycare, Runcorn, United Kingdom

## Abstract

**Aims:**

The purpose of this QIP was to increase the number of patients who had had the opportunity to discuss nicotine replacement therapy (NRT) with a member of the clinical team. This was to try and improve the rate of NRT prescriptions and to ensure the ward was aligning its’ care with the trust values of treating the patient as a whole person and focusing on patient’s total health and wellbeing needs and with being a smoke-free site.

**Methods:**

Data for all 17 beds on the inpatient ward was collected from 14/10/25–01/12/25.The inclusion criteria were any current inpatients who were current smokers, non-smokers were excluded. A spreadsheet containing columns for the bed number, smoker/non-smoker, recorded conversation about NRT, NRT prescription, and options for long-acting and short-acting NRT, was used to collect data. Data was collected initially to obtain a baseline, then re-collected following the implementation of ward-based interventions.

Interventions to improve the number of conversations about and prescriptions of NRT included discussing this in the daily morning MDT meeting to improve awareness, putting posters about NRT in the ward and doctor’s office and adding a column for NRT to the patient list in the nursing station.

**Results:**

In the initial data collection, 15 patients were recorded. 1 of these patients was a non-smoker so was excluded. NRT conversations were only documented in 2 out of the 14 patients (14.3%). NRT was prescribed in 3 out of 14 patients (21.4%).

In the post-intervention data collection, 16 patients were recorded. 4 of the 16 patients were non-smokers and excluded. Documented NRT conversations occurred in all 12 patients (100%), however 0 of these patients had NRT prescribed due to declining the intervention.

**Conclusion:**

Following the introduction of ward-based interventions, documentation of NRT conversations improved from 14.3% to 100%. This represents a significant improvement. However, this did not translate into increased NRT prescribing. In fact, there were fewer patients prescribed NRT in the second data collection due to patients declining the intervention. This suggests that in future, there could be a focus on understanding the barriersto uptake. Through strengthening patient education, we can help ensure that these conversations translate into increased NRT use, with a long-term aim of smoking cessation.

